# Cotherapy of Tiron and selenium against vanadium induced toxic effects in lactating rats

**Published:** 2011

**Authors:** Sadhana Shrivastava, Deepmala Joshi, Monika Bhadauria, Sangeeta Shukla, Ramesh Mathur

**Affiliations:** UNESCO Satellite Center of Trace Element Research and Reproductive Biology and Toxicology Laboratory, School of Studies in Zoology, Jiwaji University, Gwalior (MP), 474 011, India.

**Keywords:** *Vanadium*, *selenium*, *Suckling*, *lactating*, *Teratogenecity*, *Tiron*

## Abstract

**Background:**

Vanadium is an important environmental and industrial pollutant. It has a status of reproductive toxicant and is reported to cross placental barrier.

**Objective:**

The current study was performed to assess the therapeutic efficacy of Tiron and its combination with selenium against vanadium induced toxicity in lactating and suckling rats.

**Materials and Methods:**

Rats were exposed to vanadium at a dose of 7.5 mg/kg/day (p.o.) for 20 days from 0 day of post partom (p.p.). Tiron (606 mg/kg/day, i.p.) and selenium (0.5 mg/kg/day, p.o.) were administered for 5 days on 21-25 day PP.

**Results:**

Vanadium exposure decreased blood sugar level while serum transaminases and serum alkaline phosphatase showed increased values significantly (p<0.01). Elevation in glycogen content of liver and kidney of suckling and kidney of lactating rats was found after toxicant administration. Toxicant intoxication increased the enzymatic activity of acid phosphatase in liver of suckling and lactating and kidney of suckling rats. On the contrary alkaline phosphatase and adenosine triphosphatase activities were inhibited significantly (p<0.01) in all the organs. Lipid peroxidation was enhanced whereas glutathione was reduced significantly in liver of suckling and lactating rats (p<0.01). Vanadium also caused histopathological lesions. Therapies of Tiron per se and Tiron along with selenium maintained almost all blood and tissue biochemical parameters towards normal. Tiron along with selenium reduced vanadium induced lesions in lactating and sucklings rats.

**Conclusion:**

Tiron along with selenium is more effective than Tiron alone against vanadium induced toxic effect on lactating and suckling rats.

## Introduction

Industrial pollution is the biggest health hazard in the 21^st^ century. Vanadium is widely distributed in the earth’s crust in a wide range of minerals and in fossil fuels. The chemical gets into the air, water and soil when fuel oil is burned, or when rocks and soil containing vanadium are broken down (1-3).

It has been recognized as essential nutrients in higher animals (4). 

It has a status of reproductive toxicant, as microtubule damaging agent, reducing sperm motility and damaging spermatozoa. Vanadium decrease fertility and after expose to it, embryo lethality, fetotoxicity and teratogenicity have been reported to occur in rats, mice and hamsters (5-8). It also increases malformations and abnormalities in pups. It can pass the blood placenta barrier and has been reported to be teratogenic in rodents and affects sexual development in pre-pubertal animals. 

The development of the offspring was significantly decreased from birth and during the lactation period after vanadium intoxication (9, 10). 

Tiron, 4, 5-dihydroxy-1, 3-benzene disulfonic acid, has been known to be a widely used antioxidant to rescue ROS-evoked cell death and a non-toxic chelator to alleviate an acute metal overload. It is a substrate in several enzyme reactions and has small size which facilitates cell entry and modulates intracellular electron transfer reactions as an antioxidant by scavenging free radicals (11, 12). It is also effective against various metal intoxications of beryllium, aluminium, lead and vanadium (13-16). Selenium (Se) is a trace mineral and is needed in small amounts for good health. It acts as a growth factor and is a powerful antioxidant. It is a component of selenocysteine and selenomethionine and cofactor for reduction of antioxidant enzymes such as glutathione peroxidases (17). Se protects toxic effects of metals such as cadmium, and mercury, aluminium (18-20). Se protects neuronal cells against neurotoxic effects of vanadium (21). Thus the aim of this study was to evaluate Tiron+Se as an antidote against vanadium in sucklings and lactating rats.

## Materials and methods

Detoxification of vanadium in rats. This study comes under preclinical studies on laboratory animals.

**Chemicals**

Vanadium sulphate and sodium selenite procured from Glaxo Laboratory Ltd., Bombay, India and sodium-4, 5-dihydroxybenzene-1, 3-disulfonate (Tiron) procured from Sigma-Aldrich. Other analytical grade laboratory reagents were procured from Merck (Germany) and Glaxo chemical (India).

**Animals**

Female albino rats of Sprague Dawley strain (16010 g b.w.) from our departmental animal facility were given a standard pellet diet (Pranav Agro Industries, New Delhi, India having metal contents in ppm dry weight Cu 10; Mn 33; Zn 45; Co 5). 

Drinking water was provided *ad libitum*. Animals used in this study were treated and cared for in accordance with the guidelines recommended by the Committee for the Purpose of Control and Supervision of Experiments on Animals (CPCSEA), Government of India, Ministry of Culture, Chennai. The dose of the chelating agent was prepared daily and its pH was adjusted to 6.4 with sodium bicarbonate before administration.

**Experimental design**

Animals were selected on day 1 post partum and were divided into four groups of six animals each. Group 1 served as control. Groups 2, 3 and 4 were the experimental groups and received VOSO_4_ (7.5 mg/kg, p.o.) for 20 days. Group 2 served as experimental control. Animals of group 3 were taken individual treatment of Tiron (606 mg/kg, i.p.) for 5 days on days 21-25 whereas group 4 were administered combination treatment of Tiron (as in group 3)+ Se (0.5 mg/kg, p.o., through catheter) for 5 days on days 21-25. Rats were observed daily for mortality and morbidity through out the study. 24 h after the final treatment animals were sacrificed under light ether anesthesia. The lactating and sucklings rats were examined for any external and pathological lesions. The lactating’s blood, liver, kidney, uterus and ovary were investigated. Liver and kidney of suckling’s were also processed for biochemical assays and histopathological observation. 

**Biochemical assays**

Blood was collected and serum was isolated for various blood biochemical assays directly from the heart by puncturing the retro-orbital venous sinus. Blood sugar (22) aspartate aminotransferase (AST), alanine aminotransferase (ALT) (23), serum alkaline phosphatase (SALP) (24) and serum protein (25) were studies. Standard techniques were used to determine glycogen contents in fresh tissue (26). A homogenate in isotonic solution was processed for the determination of protein (25) activities of acid and alkaline phosphatases (ACP and ALP) (24) and adenosine triphosphatase (ATP) (27). Lipid peroxidation (LPO) (28) and reduced glutathione (GSH) (29) were also estimated. 

**Histopathology**

Liver, kidney, uterus, and ovary of lactating rats and suckling’s liver and kidney were dissected out, washed in saline and fixed in Bouin’s fluid, embedded in paraffin, sectioned at 6μm and stained with haemotoxylin and eosin for light microscopical study.

**Statistical analysis**

Data were expressed as mean ± standard error of mean (SEM). Statistical comparisons between all groups were performed by using one way ANOVA followed by student’s t-test. P-value was taken as significant at 0.01% level (30).

## Results

**Biochemical observation**

Table I illustrates the protective effect of Tiron and its combination with Se against vanadium toxicity. Vandium induced significant elevation in the activities of AST, ALT, SALP and serum protein contents whereas the level of blood sugar was decline significantly (p<0.01) in lactating rats. 

Combination therapy (T+Se) effectively recouped these values in all the parameters compare with Tiron alone. After the injection of vanadyl sulphate the wet weight of liver, kidney, uterus, and ovary of lactatings and liver and kidney of sucklings (Data not shown) showed no significant variation. LPO showed enhance values whereas glutathione showed inhibition in liver of lactatings (p<0.01) and sucklings. Chelation therapy showed improvement (Table IV). 

Glycogen content of liver, uterus, ovary of lactatings showed decreased values while kidney of lactatings and liver and kidney of sucklings showed elevated values after vanadium exposure. With the combined treatment, values were restored towards control (Table II). After administration of vanadyl sulphate, protein content were decreased in all the organs of lactatings and sucklings (Table II). Activity of acid phosphatase showed increased level in liver of lactatings, and liver and kidneyof sucklings, while its activity decreased in uterus and kidney of lactatings. With combined treatment, values showed improvement. This improvement was more effective with the Tiron+ Se (Table IV). Activities of adenosine triphosphatase and alkaline phosphatase showed inhibition after vanadium exposure in liver, kidney, and uterus of lactatings and liver and kidney of sucklings and values restored with Tiron and Tiron+ Se. Tiron+ Se was found effective (Table III and IV).

**Histological observations**

Vanadium induced proliferation in canaliculi, hypertrophy and angular nuclei in hepatocytes and sinusoidal spaces were filled with debris (Figure 1-1). 

Tiron protects the hepatocytes (Figure 1-2). Tiron+ Se restore the structure of hepatocytes showing normal sinusoidal spaces with mitotic figures. Hepatocytes showed well-maintained cytoplasm (Figure 1-3). Glomeruli showed hypertrophy in the regions of cortex, disturbed endothelial lining and degeneration in uriniferous tubules (Figure 1-4). 

Tiron showed better organization of kidney (Figure 1-5). Treatment with Tiron+Se therapy showed well form glomeruli and renal tubules (Figure 1-6). Liver of sucklings, after injection of vanadium in lactating rats, reduced sinusoidal spaces and debris was observed in some sinuses (Figure 2-7). 

Treatment of Tiron showed normal portal triads and well formed hepatocytes (Figure 2-8). Tiron+ Se therapy maintained hepatocytes and smooth lining of sinus (Figure 2-9). 

After administration of vanadium, glomeruli occupied whole Bowman’s capsule and degeneration in proximal and cortical tubules (Figure 2-10). With the treatment of Tiron, kidney showed rounded Glomeruli with maintained tubules (Figure 2-11). 

Tiron+ Se therapy improved proximal and cortical tubules with Bowman’s capsule (Figure 2-12). Toxicant exposed rats showed disorganized uterine epithelium and uterine glands (Figure 3-13). Tiron treatment showed recoupment (Figure 3-14). 

The well formed endometrium, intact columnar epithelium, well developed musculature prominent vascularity and stroma was noted after treatment of Tiron + Se (Figure 3-15). After vanadium administration, decreased matured follicles, disintegration in ovum, disorganized and hypertrophied developing follicles were seen. Stroma was loose and fibrotic (Figure 3-16). Treatment of Tiron showed normal position of follicles along with few and atretic follicles (Figure 3-17). 

Combination therapy showed well organization of developing follicles, freshly ovulated follicles and maintain stroma (Figure 3-18).

**Table I:** Effect of Tiron (T) and selenium (Se) on liver function tests against vanadium (V) intoxication

**Treatments**	**Control**	**V**	**V+T**	**V+T+Se**	**ANOVA**
Blood Sugar (mg/100 g)	101.6+5.49	69.1+3.52#	92.5+4.91	99.8+5.04*	11.6†
AST (IU/L)	64.6+4.28	124.8+6.55#	95.2+5.46*	84.2+5.24*	25.5†
ALT(IU/L)	41.6+2.15	83.6+4.40#	69.2+4.42	50.1+2.98*	32.6†
SALP(mg Pi/100 ml/h)	207.2+10.5	463.2+30.4#	393.4+20.1	311.8+16.2*	34.1†
Serum Protein (mg/100 ml)	36.4 +2.07	70.2+3.73#	69.8+3.65	66.4+3.74	27.8†

AST = aspartate aminotransferase, ALT = alanine aminotransferase, SALP = serum alkaline phosphatase. Values are mean+S.E., (n=6), ANOVA ‘F’ values,

† = Significant at 1% level.

# p< 0.01 compared to control group,

* p < 0.01 compared to toxicant administered group.

**Table II:** Effect of Tiron (T) and selenium (Se) on protein (mg/100 mg) and glycogen (mg/100 g) contents against vanadium (V) intoxication.

**Treatments**		**Control**	**V**	**V+T**	**V+T+Se**	**ANOVA**
Sucklings						
	Protein (L)	10.76+0.66	9.86+0.50	10.60+0.53	10.70+0.79	0.52
	Protein (K)	9.23+0.66	7.42+0.41	8.61+0.46	9.58+0.46*	4.12
Lactating						
	Protein (L)	16.78+0.94	12.24+0.62#	12.94+0.67	15.16+0.92	8.00†
	Protein (K)	12.69+0.84	9.26+0.56#	9.65+0.52	9.86+0.56	7.23†
	Protein (U)	10.28+0.60	7.98+0.42	8.15+0.43	9.58+0.64	5.23†
Sucklings						
	Glycogen (L)	2726.2+153	3648.4+206#	3066.8+161	2996.4+191	5.59†
	Glycogen (K)	66.8+3.43	80.6+4.24	74.4+4.02	70.7+4.42	2.51
Lactating						
	Glycogen (L)	2892.3+151	1515.8+108#	2598.6+149*	2690.8+149*	23.0†
	Glycogen (K)	62.20+3.45	87.8+5.60#	68.6+3.55	61.2+3.12*	11.0†
	Glycogen (U)	123.4+8.28	73.5+4.12#	104.0+5.23*	118.4+8.04*	13.5†

L = Liver, K = Kidney, U = Uterus

Values are mean + S.E., (n = 6), ANOVA ‘F’ values,

† = Significant at 1% level.

# p< 0.01 compared to control group,

* p< 0.01 compared to toxicant administered group.

**Table III:** Effect of Tiron (T) and selenium (Se) on acid (ACPase) and alkaline phosphatase (ALPase) (mg Pi/100 mg/h) against vanadium (V) intoxication

**Treatments**		**Control**	**V**	**V+T**	**V+T+Se**	**ANOVA**
Suckling						
	ACPase (L)	237.2+14.9	265.4+13.7	238.7+13.5	237.2+14.8	1.12
	ACPase (K)	207.7+12.5	271.0+18.7	232.2+14.8	226.4+12.0	3.87
Lactating						
	ACPase (L)	248.1+17.7	324.6+20.9	291.3+15.9	255.6+15.4	4.76
	ACPase (K)	288.8+15.9	238.0+16.8	260.4+19.1	281.2+15.4	2.18
	ACPase (U)	183.4+11.4	73.2+4.05#	158.2+8.82*	165.8+11.7*	31.7†
Sucklings						
	ALPase (L)	80.6+4.29	57.4+3.30#	72.6+4.51	79.8+4.26*	8.19†
	ALPase (K)	2306.8+118	1378.2+68.7#	1785.8+90.7*	2009.6+112*	18.5†
Lactating						
	ALPase (L)	79.7+4.77	66.1+3.58	69.4+4.60	74.1+4.30	2.23
	ALPase (K)	2233.0+118	1046+60.4#	1636.2+109*	1892.1+98.7*	30.4†
ALPase (U)		472.9+26.0	233.6+15.0	301.6+22.0	324.2+17.2	28.8†

L = Liver, K = Kidney, U = Uterus

Values are mean + S.E., (n = 6), ANOVA ‘F’ values,

† = Significant at 1% level.

# p< 0.01 compared to control group,

* p< 0.01 compared to toxicant administered group.

**Table IV:** Effect of Tiron (T) and selenium (Se) on adenosine triphosphatase (ATPase, mg Pi/100 mg/min), Lipid peroxidation (LPO, n mole MDA/mg protein) and reduced glutathione (GSH, µ mole/g ) against vanadium (V) intoxication

**Treatments**		**Control**	**V**	**V+T**	**V+T+Se**	**ANOVA**
Suckling						
	ATPase (L)	844.4+43.1	625.8+44.8#	866.6+49.3*	880.4+52.7*	7.60†
	ATPase (K)	2284.6+138	1325.2+87.9#	2142.6+124*	2153.4+122*	16.1†
Lactating						
	ATPase (L)	1804.0+102	1357.4+ 84.0#	1698.0+85.5	1896.6+95.5*	7.80†
	ATPase (K)	2228.7+126	1570.8+85.5#	2132.4+115*	2102.0+125*	8.09†
	ATPase (U)	774.2+51.4	535.4+27.8#	543.2+27.7	739.8+40.3*	13.1†
Sucklings						
	LPO (L)	0.40+0.02	0.49+0.02	0.43+0.02	0.41+0.02	2.79
	GSH (L)	7.40+0.39	5.79+0.43	6.64+0.36	7.19+0.38	3.95
Lactating						
	LPO (L)	0.33+0.01	0.86+0.04#	0.46+0.02*	0.42+0.02*	67.50†
	GSH (L)	8.36+0.43	4.81+ 0.25#	7.64+0.40*	7.90+0.42*	20.75†

L = Liver, K = Kidney, U = Uterus

Values are mean + S.E., (n = 6), ANOVA ‘F’ values,

† = Significant at 1% level.

# p< 0.01 compared to control group,

* p< 0.01 compared to toxicant administered group.

**Figure:**
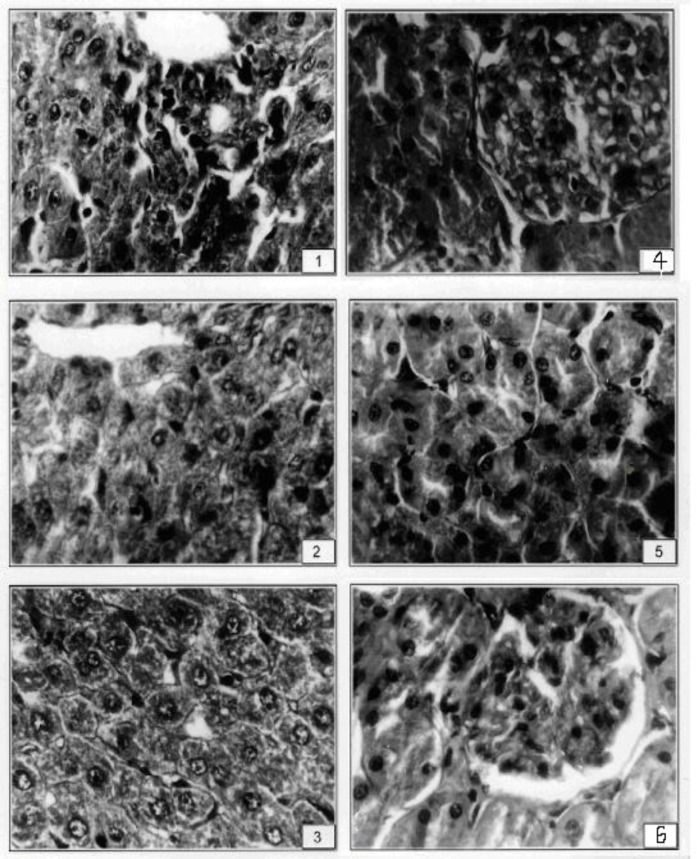
1*.* After V exposure, hepatocytes showed degeneration along with vacuolation and granulation (X 400). 2. Hexagonal hepatocytes were seen after conjoint treatment with Tiron (X 400). 3. After the treatment of T+ Se mitotic figures were seen in some hepatocytes (X 400). 4*.* After the administration of vanadyl sulphate kidney showed hypercellularity in glomeruli of Bowman’s capsule (X 400). 5. Tiron treatment after V exposure showed better organization of cortex region of kidney (X 400). 6. Conjoint treatment of T+Se showed well formed Bowman’s capsules with glomeruli (X 400).

**Figure:**
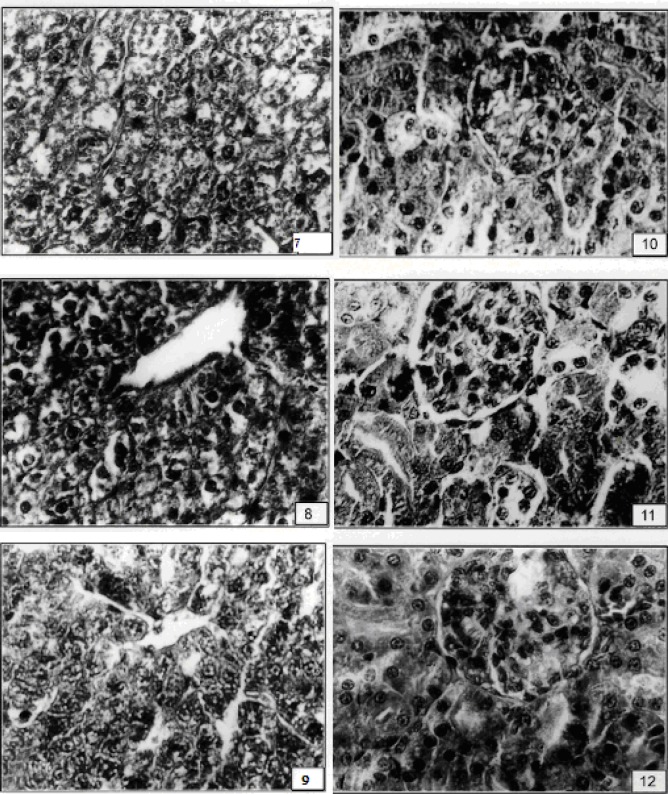
7. V caused hypertrophy in hepatocytes of liver of sucklings (X 400). 8. Hexagonal hepatocytes were seen after therapy with Tiron (X 400). 9. With the treatment of T + Se improvement was shown (X 400). 10. After administration of vanadyl sulphate kidney showed glomeruli occupied whole spaces of Bowman’s capsule (X 400). 11. Tiron treatment after vanadium exposure showed better organization of cortex region of kidney (X 400). 12. Conjoint treatment of T+ Se showed normal Bowman’s capsules with glomeruli in kidney of sucklings (X 400).

**Figure:**
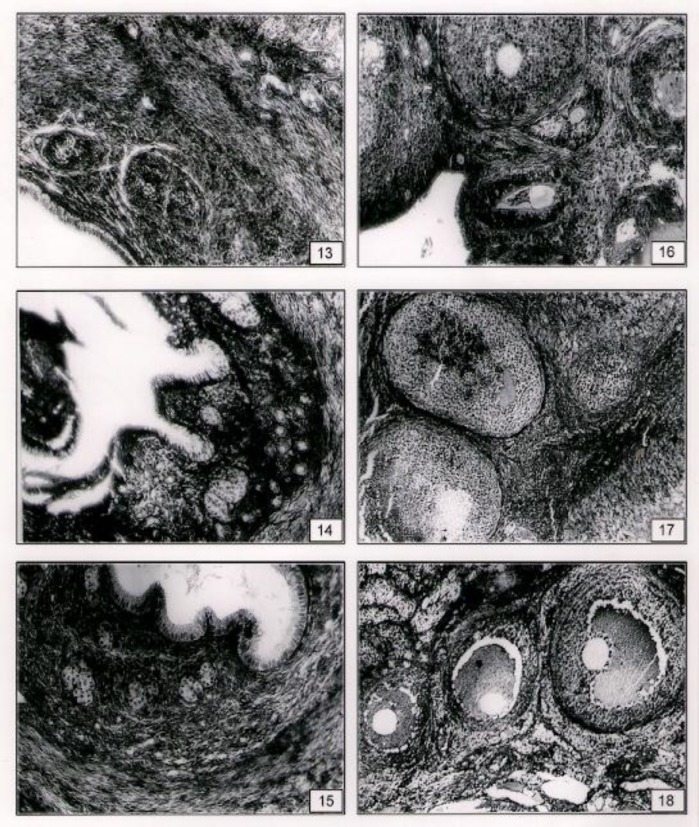
13. Vanadium exposed rats showed degenerative changes in endometrium with degenerated uterine glands (X 120). 14. With the treatment of Tiron after V exposure endometrial cells were well formed with loose stroma (X 120). 15. Well formed uterine glands were noted with the co-therapy of T+ Se (X120). 16. V exposure caused degenerated primary, secondary follicles with loose stroma in ovary (X 120). 17. Tiron treatment after V exposure showed mostly normal secondary and tertiary follicles with normal lutin cells (X 120). 18. With the treatment of T+Se after vanadium exposure better organization of the follicles were seen (X 60).

## Discussion

The efficacy of exogenous therapy of Tiron in combination with Se in the acceleration of vanadium elimination and in the reversal of intoxication has been discussed. The aim of the present study was to report the teratogenic effects of vanadium and to develop an intervention strategy of companion formula of chelators with antioxidant effects against vanadium intoxication. About 95% of the vanadium transported in the blood is bound to transferrin as the vanadyl (IV), it also complexs with lactoferrin to be transported to sucklings during breast feeding. Vanadium is present in placenta and competes with iron for both gastrointestinal absorption and cellular receptor sites (31-33). 

Research indicates that ALT and AST can be used as biomarkers of cellular damage in blood plasma, protein degradation and liver damage with loss of the functional integrity of cell membranes (34), thus a direct correlation exists between rise in the serum enzyme activity and severity of damage. 

The ALT is localized mainly in the cytoplasm whereas AST activity is fairly distributed between cytoplasm and mitochondria. A significant increase in AST, ALT and SALP activities was noted after vanadium exposure in lactating rats. The elevated activities are indicative of cellular leakage and loss of the functional integrity of cell membranes, thus necrosis or membrane damage which releases the enzyme into circulation. 

SALP mainly arises from the lining of the canaliculi and also from the sinusoidal surface of hepatocytes and is excreted via bile. The SALP is closely connected with proximal tubules and osteoblast and is involved in the active transport across the capillary walls. The increased level of SALP may be due to the *de novo* synthesis by the liver cells. 

Results of the present study clearly depicted that vanadium administration enhanced concentration of this soluble enzyme significantly (34). An increase in the activities of these enzymes is supported by various authors (34, 35). 

The stabilization of AST, ALT and SALP levels by therapy of chelating agent with and without antioxidants is a clear indication of improvement in the functional status of the liver cells (13, 14). 

Oral administration of Se attenuated significantly the increased level of these enzymes and caused a subsequent recovery towards normalization, as seen from statistical analysis. Combined therapy may combine with reactive metabolites and lead to inactivate them, thus it may prevent the acute organ dysfunction and cellular injury thereby inhibiting the rapid leakage of these enzymes. 

A number of investigators have previously demonstrated the antioxidative effect of magnesium (36), zinc (37) and selenium (20) etc. Activities of these enzymes were also recouped significantly with the administration of Tiron against other metals, such as, beryllium (13), aluminum (14) and vanadium (16, 35). Lowering of blood sugar level in the lactating rats may be due to increase in the uptake of glucose in the glycogen metabolism (34). In vanadate treated rats, liver glycogen level elevated in sucklings whereas decreased in lactating animals as seen in the present study. This can be very well correlated with the increased activity of glycogen synthetase and decreased activity of glycogen phosphorylase (38). 

Improvement in blood sugar and serum protein by Tiron treatment is also reported (13, 34). Oxidative stress is a major pathway for vanadium induced toxicity (39). The pro-oxidant effect of vanadate was evident in our experiments by the occurrence of LPO in liver (2, 40). A significant increase in malaondialdehyde products was also observed after exposure to metals such as mercury (19), aluminium (14, 20) and beryllium (13). GSH plays a major role in cellular defense and in the maintenance of the thiol disulfide status. It showed inhibition in liver of sucklings and lactating rats because of bonding to sulfhydryl groups of proteins. 

The increased TBARS as seen in the present study may be due to tissue injury and failure of antioxidant defense mechanism. Therefor increased accumulation of LPO products might well be the consequences of a progressive degradation of necrotic tissue. LPO mainly damages Kupffer cells as also evident in histological studies. The result suggests that toxicant induced elevation in LPO might be because of the lower level of GSH as observed in this study. GSH depletion decreases the GSH/GSSG ratio, which leads to the production of free radicals (41). 

Combined therapy clearly indicated the supplementation of antioxidants increased effectiveness of Tiron which help to regenerate and maintain the normal functional status of the tissues. Treatment with Se afforded better protection, this may be due to the destruction of free radicals, supplying a competitive substrate for unsaturated lipids in the membrane and/or accelerating the repair mechanism of damaged cell membrane. Therefor combination therapy (Tiron+ Se) effectively recouped these values than tiron per se. These findings are substantiated by various studies (13, 14, 42). 

Significant decreased values were seen in ALPase, ATPase and increased value noted in ACPase due to the vanadyl ion formed may bind with cell proteins. VO^2+ ^resembles with size of Mg^2+^ which is regulated for the activity of ALPase (21, 33), thus was inhibited due to its structural similarity with phosphate, vanadate (VO_3_) also interferes with a large variety of phosphate dependent enzymes (43). Vanadate is a potent inhibitor of membrane bound ATPase (33). It is also possible that accumulation of vanadium with its concomitant reduction to vanadyl followed by damage to biological membranes, lysosomal enzymes releases and destruction of placental tissue (34). 

Fatty changes with partial cell necrosis in the liver have been observed in rats as a result of exposure to vanadium (34). In the present study vanadium induced; proliferation in canaliculi, hypertrophy and angular nuclei in hepatocytes, hypertrophy in glomeruli and degenerated uriniferous tubules, disorganized uterine epithelium and disintegration in ovum. Vanadium was accumulated within spheroidal electro dense structures in the cytoplasm of these cells (44- 46). Therapy with Tiron was found very effective and this may be due to its diphenolic nature of Tiron which forms water soluble complexes with a large number of metals (13, 14). Thus, it is clearly apparent that Tiron crosses the placental barrier as reported for other metals as well (42). Se acts as cofactor of many antioxidant enzymes, maintains the availability of antioxidant nonprotein sulfhydryl groups (47, 48) thus may induce binding of the V-Se complexes to proteins. It is an important nutrient and can pass through the milk, thus ameliorating toxicity in lactating and sucklings. 

In conclusion, the present research has identified the role of antioxidants, Se and Tiron in mitigation of vanadium toxicity by acting synergistically. 
